# Integrated Analysis of Genomic and Transcriptomic Profiles Identified the Role of GTP Binding Protein-4 (GTPBP4) in Breast Cancer

**DOI:** 10.3389/fphar.2022.880445

**Published:** 2022-06-16

**Authors:** Yiming Hu, Jiaheng Xie, Liang Chen, Qikai Tang, Wei Wei, Wenfeng Lin, Wang Du, Tinghong Xiang, Lu Yin, Jing Ji

**Affiliations:** ^1^ College of Pharmacy, Jiangsu Ocean University, Lianyungang, China; ^2^ Department of Burn and Plastic Surgery, The First Affiliated Hospital of Nanjing Medical University, Nanjing, China; ^3^ Department of General Surgery, Fuyang Hospital Affiliated to Anhui Medical University, Fuyang, China; ^4^ Department of Neurosurgery, The First Affiliated Hospital of Nanjing Medical University, Jiangsu Province Hospital, Nanjing, China; ^5^ Department of Cardiovascular Surgery, The First Affiliated Hospital of Nanjing Medical University, Nanjing, China

**Keywords:** bioinformatics, breast cancer, Tumor-infiltrating immune cells, prognosis, biomarkers

## Abstract

**Purpose:** To explore the significance of GTP-binding protein 4 (GTPBP4) in breast cancer.

**Methods:** Firstly, GTPBP4 expression analysis was performed in TIMER and UALCAN databases. Subsequently, the TCGA cohort and multiple Gene Expression Omnibus Cohorts were used as validation for GTPBP4 expression. Besides, we also evaluated the diagnostic value of GTPBP4 in TCGA Cohort and multiple GEO Cohorts. The predictive effect of GTPBP4 in breast cancer was then assessed using survival analysis. Then we look at the role of GTPBP4 in the immune milieu and create a Nomogram to help patients with breast cancer understand their prognosis. Finally, *in vitro* tests were carried out to look at GTPBP4 expression and function in breast cancer cell lines.

**Results:** GTPBP4 is an independent breast cancer prognostic factor that is upregulated in the disease (*p* < 0.05). Enrichment analysis showed that GTPBP4 was associated with multiple functions and pathways. In addition, GTPBP4 is associated with a variety of immune cell types (*p* < 0.05). PCR assay showed that GTPBP4 expression was up-regulated in breast cancer cell lines. The activity, migration, and proliferation of breast cancer cells were considerably reduced after GTPBP4 knockdown in the CCK-8, Transwell, and Scratch assays.

**Conclusions:** Our research discovered a new breast cancer biomarker that can be used as a guide for breast cancer diagnosis and treatment.

## Introduction

Breast cancer (BRCA) has surpassed lung cancer as the most frequent type of cancer worldwide, according to a WHO report released in early 2021 (Seale and Tkaczuk, 2022). Breast cancer is the leading cause of death among women, and the situation is still dire (Libson and Lippman, 2014; Odle, 2017). Although breast ultrasound, mammography, MRI, and other examinations can enable some breast cancer patients to be diagnosed relatively early, there are still a considerable number of patients, especially in less developed areas, who are delayed diagnosed, resulting in adverse consequences (DeSantis et al., 2011; Woolston, 2015; Peairs et al., 2017; Xie et al., 2021a). Sequential therapies such as neoadjuvant chemotherapy, targeted therapy, and endocrine therapy have also improved the prognosis of breast cancer patients (Kolak et al., 2017). But for triple-negative breast cancer or advanced breast cancer, the effect is still not ideal (Menta et al., 2018). As a result, new biomarkers must be investigated in order to assess the prognosis of breast cancer patients and develop new treatment options.

GTP binding protein (GTPBP) is a kind of GTP enzyme with a molecular switching function (Yu et al., 2016). When binding to GTP, the protein is activated to regulate a cascade of downstream pathways (Maiti et al., 2021). When GTP is hydrolyzed into GDP, the protein’s function is temporarily halted. GTPBP4 is a member of the protein family (Maiti et al., 2021). The importance of GTPBP4 in several malignancies has only recently been discovered. GTPBP4 can enhance the growth of gastric cancer via modulating the activity of p53, according to Li et al. (Li et al., 2018). GTPBP4 knockdown decreased the proliferation of hepatocellular carcinoma (HCC) cells, according to Liu et al. (Liu et al., 2017). Up-regulation of GTPBP4 expression was linked to lymph node metastasis and improved HCC invasion ability, according to Chen et al. (Chen et al., 2021a). In conclusion, GTPBP4 has a lot of potential in cancer. GTPBP4’s significance in breast cancer, however, is unknown.

At present, with advances in computer technology and the development of public databases, it is possible to explore cancer genomics by using sequencing data and clinical information in public databases (Xie et al., 2021b). The Cancer Genome Atlas (TCGA) database and the Gene Expression Omnibus (GEO) database are two of the most widely utilized bioinformatics databases (Chen et al., 2021b). We can undertake a wide range of genomic analyses using these public databases, including expression analysis, survival analysis, and immunological microenvironment analysis (Li et al., 2021).

In this study, the expression, prognostic significance, and immunological connection of GTPBP4 in breast cancer were investigated. Our findings could lead to new approaches to breast cancer treatment.

## Materials and Methods

### Expression Pattern of GTP Binding Protein4 in Breast Cancer Based on GEPIA and TIMER Databases

The UALCAN Database (http://ualcan.path.uab.edu) is a web-based tool for analyzing differential expression and survival data from the Cancer Genome Atlas (TCGA) and Genotypic Tissue Expression (GTEx) projects. The TIMER database (http://cistrome.dfci.harvard.edu/TIMER/) is a tool for analyzing differential expression and immune infiltration. GTPBP4 expression variations between BRCA and normal tissues were detected using these two online databases (UALCAN, TIMER).

### The Cancer Genome Atlas and Gene Expression Omnibus Data Download and Processing

From the TCGA and GEO databases, we collected BRCA datasets with GTPBP4 sequencing data. First, the TCGA database was used to retrieve gene expression and clinical data from BRCA patients. After that, log2 conversion to RNAseq data in FPKM (Fragments Per Kilobase Per Million) format is conducted. The GEO database’s BRCA datasets were then used for validation. The GEO datasets used in this study must adhere to the following guidelines: 1) The species is *Homo sapiens*; 2) The data comprised the expression profile of GTPBP4 in BRCA; 3) The total number of samples in both the tumor and non-tumor groups was more than 10. All data from the GEO database has been normalized and log2 transformed. Based on R software, ggplot2 package was used to visualize GTPBP4 expression.

#### The Receiver Operating Characteristic Curve of GTPBP4 in Each Cohort was Constructed to Explore its Diagnostic Sensitivity

The receiver operating characteristic (ROC) curves related to GTPBP4 were constructed in TCGA and multiple GEO datasets. The area under the curve (AUC) was used to calculate GTPBP4’s diagnostic sensitivity (AUC).

### Comprehensive Analysis of GTPBinding Protein4 in Multiple Gene Expression Omnibus Datasets

To reduce the impact of random error, the meta-analysis combined GTPBP4 expression levels from the tumor group and the normal group from each independent GEO data set. The mean ± standard deviation of each data set was calculated first. The combined SMD (standardized mean difference) and 95 percent confidence interval (CI) were then calculated. The expression level of GTPBP4 in BRCA was substantially higher than that in the normal group if SMD>0 and *p* < 0.05 were detected. We created the SROC (Summary Receiver Operator Characteristic) curve and evaluated the area under the curve (AUC) to further examine GTPBP4’s sensitivity in diagnosing BRCA.

Furthermore, the variability of particular research was investigated. If I^2^ is more than 50%, the random-effects model is used. The fixed effects model is utilized if I^2^ is less than 50%. Finally, to assess publication bias, the Begg and Egger tests were used. There was no significant publication bias in this study when *p* > 0.05. Begg’s test, Egger’s test, and SROC analysis were done with Stata program, while other analyses were done with R software’s “meta” and “forestplot” packages.

### Prognostic Value of GTPBinding Protein4 in Breast Cancer

The TCGA cohort was then used to investigate the predictive usefulness of GTPBP4 in breast cancer. TCGA patients were separated into two groups based on the median value of GTPBP4 expression: high expression and low expression, with 535 patients in each group. The “survival” package in R was then used to investigate survival differences between the two groups. To see if GTPBP4 was an independent predictive marker for breast cancer, researchers used univariate and multivariate COX regression. Statistical significance was set at *p* < 0.05.

### Gene Ontology Enrichment Analysis and Gene Set Enrichment Analysis (Gene Set Enrichment Analysis)

Then, using the R software package ClusterProfiler (version 3.0.4), differential genes in high- and low-expression groups were analyzed for gene ontology (GO) enrichment, and the pathway and ontology of substantial enrichment and linked genes were characterized. The gene set enrichment analysis (GSEA) was the next step in the enrichment analysis process. GSEA starts with a set of biologically significant genes (such as those in a pathway), calculates the genes in the set (all of which have the same meaning/function), and then summarizes them into a single enrichment score. This method of analysis adds interpretability and was used in this study to evaluate changes in pathway/functional activity of the gene set and select the gene set with a *p* < 0.05.

### Analysis of Immune Microenvironment

We investigated differences in immune cell infiltration levels between the high GTPBP4 expression group and the low GTPBP4 expression group using the “CIBERSORT” code data and 22 immune cell comparison files on the CIBERSORT website. Then, we tested the correlation between these 22 immune cells and GTPBP4. Finally, through the intersection of different immune cell types and significantly correlated immune cell types, we obtained the most significant GTPBP4-related immune cell type in breast cancer.

### Construction of a Nomogram Based on GTPBinding Protein4 Expression

We built a nomogram model incorporating GTPBP4 expression and clinical features in the TCGA cohort to further evaluate breast cancer patient survival. To integrate GTPBP4 expression data with clinical characteristics, the “Regplot” software was used to plot patient “TCGA-AR-A255.” To assess the correctness of this Nomogram, 3- and 5-year ROC curves as well as calibration curves were created.

### Cell Lines, Culture Conditions and Cell Transfection

The Chinese Cell Repository provided MDA-MB-231, BT-549, SUM1315MO2, and ZR-75–1 breast cancer cell lines as well as human breast epithelial cell lines (HBL-100) (Shanghai, China).

All cells were grown at 37°C with 5% CO_2_ in DMEM (Gibco) with 10% fetal bovine serum (Gibco) and 1% penicillin-streptomycin solution (Gibco). These cells were transfected using the Lipofectamine3000 (Thermo Fisher Scientific, Waltham, MA, United States) according to the manufacturer’s procedure with previously generated short interfering RNAs (Hippobiotec, Huzhou, China) targeting gene GTPBP4. Supplementary Table S1 lists the siRNA sequences for the gene GTPBP4. All data were presented as the means ± SD of three independent experiments.

### Quantitative Real-Time Polymerase Chain Reaction (qRT-PCR)

Total cellular RNAs were extracted according to the manufacturer’s instructions using Trizol Reagent (Invitrogen, Carlsbad, CA, United States). PrimeScript TM RT reagentKit was used to make cDNAs (Takara, Dalian, China). On an ABI Stepone plus PCR equipment, qRT-PCR was performed using AceQ Universal SYBR qPCR Master Mix (Vazyme, Nanjing, China) (Applied Biosystems, FosterCity, CA, United States). The 2^−ΔΔCt^ technique was used to determine relative quantification. The level of glyceraldehyde-3-phosphate dehydrogenase (GAPDH) mRNA was used to standardize the relative expression of messenger RNA (mRNA) for each gene. In Supplementary Table S2, the primer sequences are listed. All data were presented as the means ± SD of three independent experiments.

### CCK-8 Assay

Cell proliferation was measured using the Cell Counting Kit-8 (CCK8) technique. A 96-well cell culture plate was used to seed the cells. Each well of the plate received 10 μL of CCK-8 solution (Biosharp, Hefei, China). The plate was then placed in the cell culture incubator for 3 h, away from light. Finally, the absorbance of each well was measured at 450 nm using a microplate reader. All data were presented as the means ± SD of three independent experiments.

### Transwell Assay

The migration of ZR-75-1 and MDA-MB-231 cells was assessed using a transwell assay. Cells were seeded into the upper well for 36 h after transfection and allowed to invade through the transwell plate. Methanol was used to fix the cells on the inserts, which were then stained with crystal violet and counted under a light microscope. All data were presented as the means ± SD of three independent experiments.

### Wound Healing Assay

Transfected ZR-75-1 and MDA-MB-231 cells were plated in 6-well plates and wounds were made with a pipette tip. Cell migration was assessed by measuring wound closure at 0 and 24 h. All data were presented as the means ± SD of three independent experiments.

### Statistical Analysis

Differences between normal tissue and tumor samples were analyzed using the Wilcoxon rank sum test. For survival analysis, Cox analysis and the K-M curve were utilized. For drug sensitivity correlation analysis, the Pearson correlation test was performed. The signature’s accuracy was assessed using a ROC curve. In the absence of special instructions, the test level was set at *p* < 0.05. The platform for analysis is R software (4.1.2).

## Results

Figure 1

**FIGURE 1:**
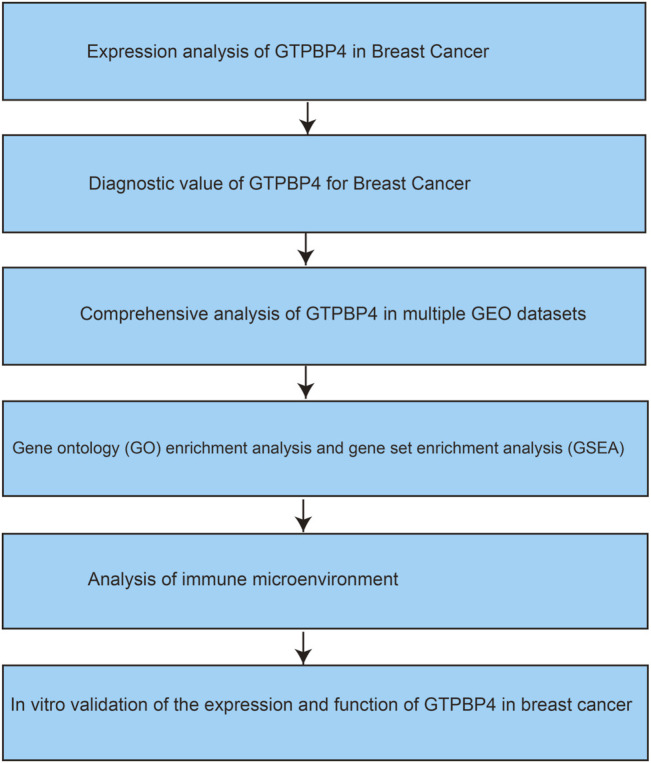
The flow chart of our study.

### Depicted the Flow Chart of Our Work

#### Expression Analysis of GTPBinding Protein4 in TIMER and UALCAN Databases

**FIGURE 2:**
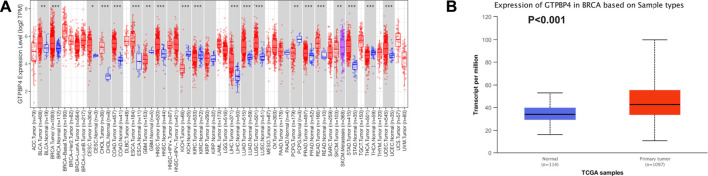
Analysis of GTPBP4 expression in online cancer databases. **(A)** GTPBP4 was differentially expressed in many cancer types in the TIMER database. Among them, GTPBP4 expression was up-regulated in breast cancer (*p < 0.05, **p < 0.01. * * *p < 0.001) **(B)** The expression of GTPBP4 in breast cancer was analyzed in UALCAN database. GTPBP4 expression was up-regulated in breast cancer compared with normal tissues (*p* < 0.001).

To begin, we analyzed the TIMER and UALCAN databases to see if there was a difference in GTPBP4 expression between breast cancer and normal tissues. The TIMER database revealed that GTPBP4 expression was up-regulated in breast cancer relative to normal tissue (*p* < 0.001), as illustrated in Figure 2A. Figure 2B showed that GTPBP4 expression was elevated in breast cancer relative to normal tissue (*p* < 0.001), as shown by the UALCAN database. Supplementary Figure S1 showed the correlation analysis of GTPBP4 with the age and stage of breast cancer patients in the TCGA cohort.

### Validation of the Expression of GTPBinding Protein4 in The Cancer Genome Atlas and Gene Expression Omnibus Cohorts

**FIGURE 3:**
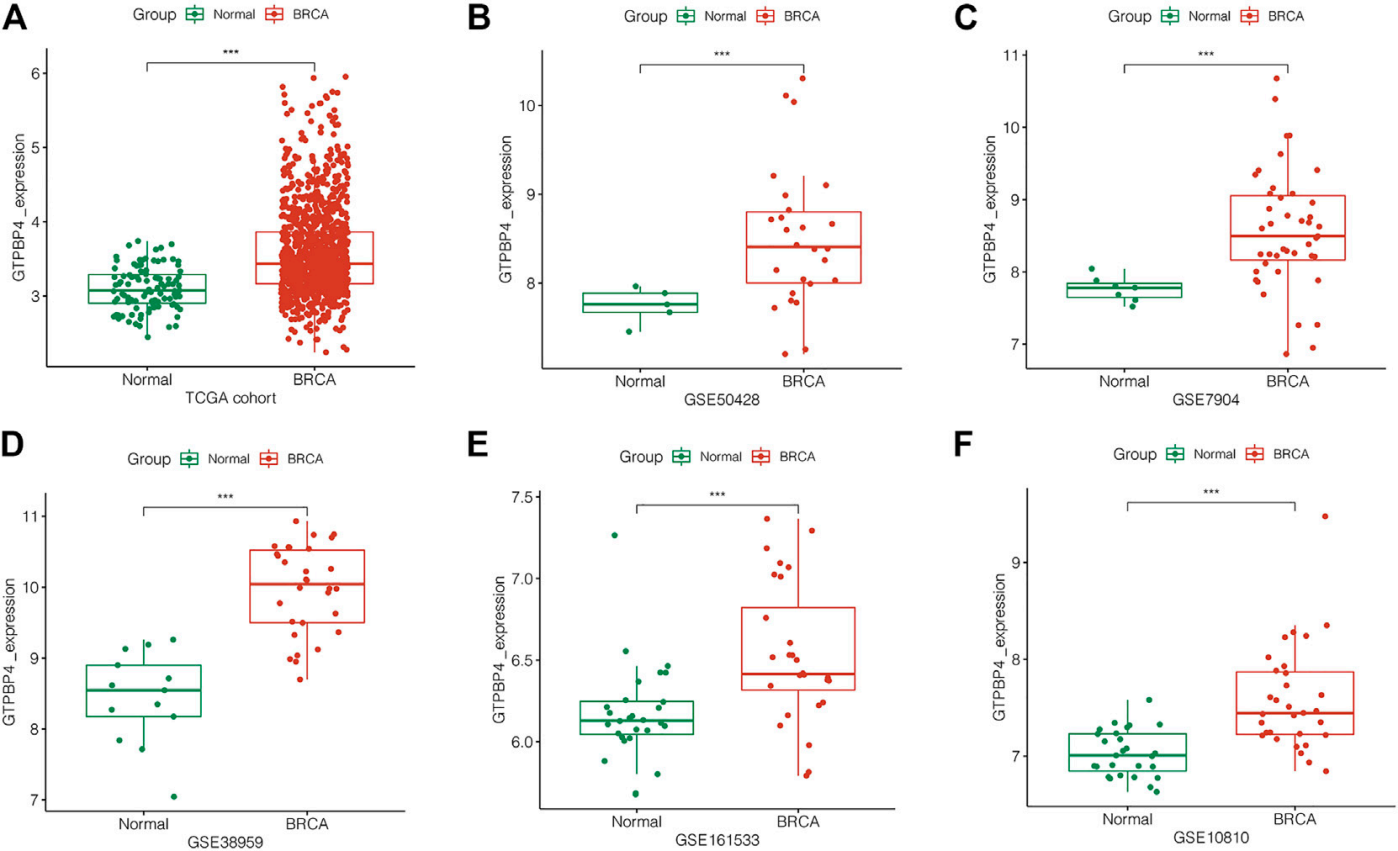
Analysis of GTPBP4 expression in TCGA and GEO cohorts. In each cohort, compared with normal tissue, GTPBP4 expression in breast cancer were significantly upregulated (***p<0.001). **(A)** TCGA cohort. **(B)** GSE50428. **(C)** GSE7904. **(D)** GSE38959. **(E)** GSE161533. **(F)** GSE10810.

To confirm GTPBP4 expression in breast cancer, we looked at it in multiple independent cohorts, including one TCGA cohort and five GEO cohorts. GTPBP4 expression was elevated in breast cancer in each independent cohort (*p* < 0.001), as seen in Figures 3A–F.

### Diagnostic Value of GTPBinding Protein4 for Breast Cancer

**FIGURE 4:**
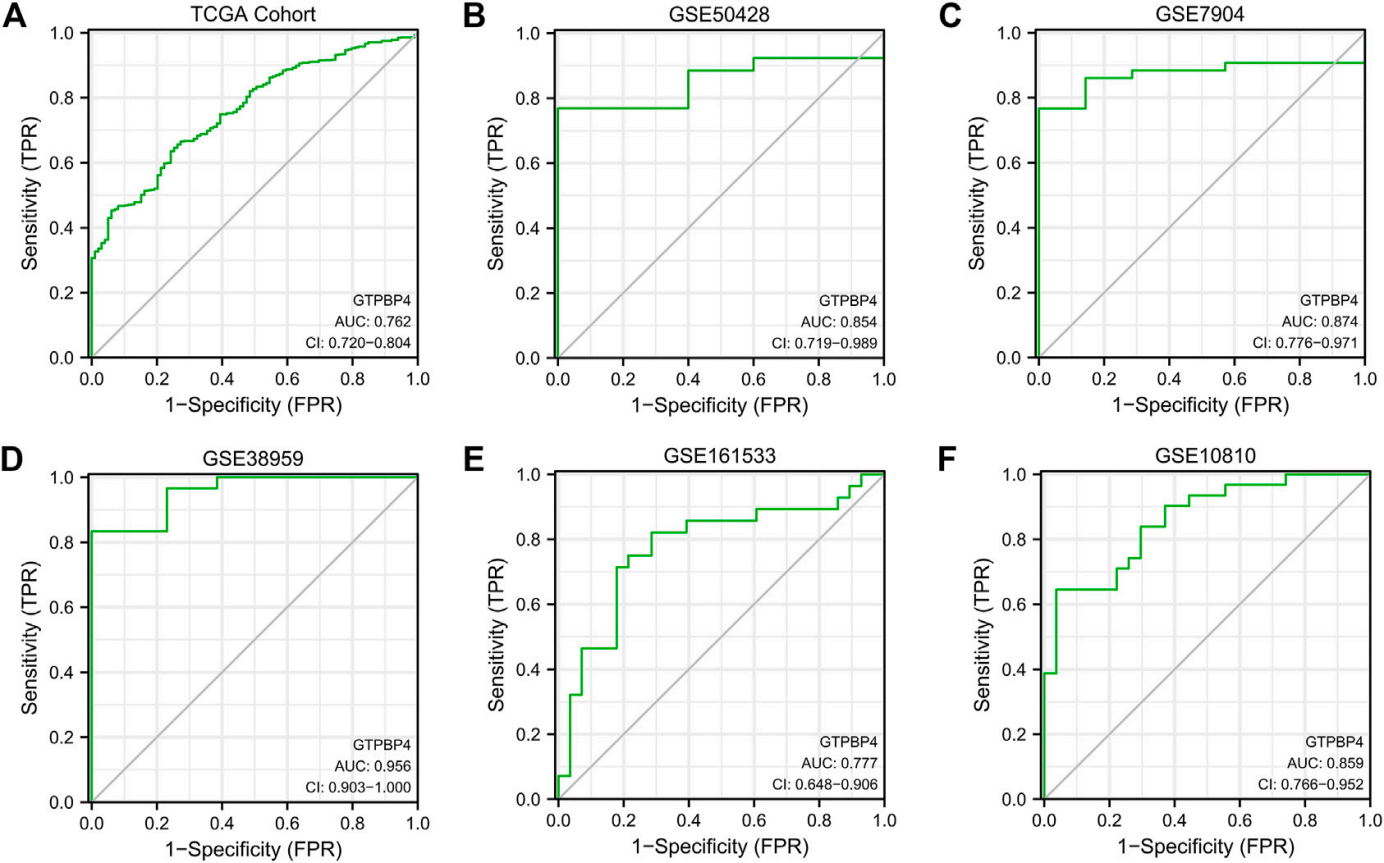
The diagnostic accuracy of GTPBP4 was quantified by constructing a ROC curve and calculating the area under the curve. **(A)** ROC curve of TCGA cohort. The AUC value is 0.762. **(B)** ROC curve of GSE50428. The AUC value is 0.854. **(C)** ROC curve of GSE7904. The AUC value is 0.874. **(D)** ROC curve of GSE38959. The AUC value is 0.956. **(E)** ROC curve of GSE161533. The AUC value is 0.777. **(F)** ROC curve of GSE10810. The AUC value is 0.859.

Following that, we built ROC curves in the 6 cohorts mentioned above, including 1 TCGA cohort and 5 GEO cohorts, to determine the diagnostic utility of GTPBP4 for breast cancer. The area under the curve (AUC) is then calculated. The AUC for the TCGA cohort is 0.762, while the AUCs for the five GEO cohorts were 0.854, 0.874, 0.956, 0.777, and 0.859, respectively (Figures 4A–F). GTPBP4 has a high diagnosis accuracy for breast cancer, according to these findings.

### Comprehensive Analysis of GTPBinding Protein4 in Multiple Gene Expression Omnibus Datasets

**FIGURE 5:**
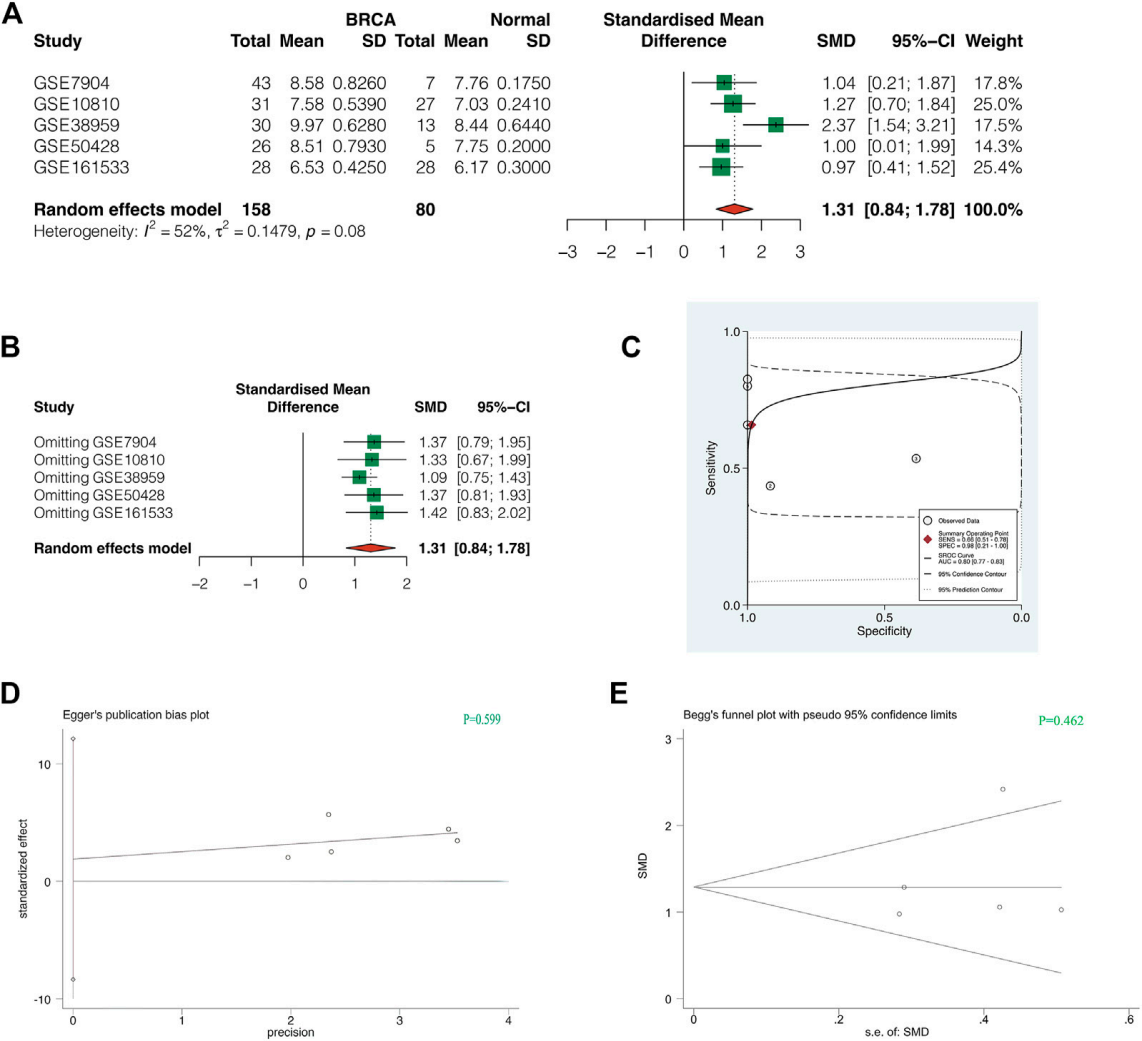
Comprehensive analysis of GTPBP4 in multiple GEO datasets. **(A)** The random effect model was used for analysis due to heterogeneity I^2^>50%. The combined SMD was 1.31, 95%CI was [0.84, 1.78]. **(B)** Sensitivity analysis showed that the results of our meta-analysis were reliable. **(C)** The AUC value of sROC curve was 0.80, indicating good diagnostic accuracy of GTPBP4 **(D,E)** The *p*-value of Egger test was 0.599, and the *p*-value of Begg test was 0.462, indicating that there was no obvious publication bias.

To increase the credibility of the results, we conducted a meta-analysis of GTPBP4 expression in 5 GEO Cohorts. As shown in Figure 5A, the random effect model was used for analysis due to heterogeneity I^2^>50%. The combined SMD was 1.31, 95%CI was (0.84, 1.78). GTPBP4 expression upregulation in breast cancer is thus plausible. Sensitivity analysis revealed that the findings of our meta-analysis were trustworthy (Figure 5B). The AUC value of sROC curve was 0.80, indicating good diagnostic accuracy of GTPBP4 (Figure 5C). Finally, the *p*-value of Egger test was 0.599 (Figure 5D), and the *p*-value of Begg test was 0.462 (Figure 5E), indicating that there was no obvious publication bias.

### The Prognostic Significance of GTPBinding Protein4 in Breast Cancer

**FIGURE 6:**
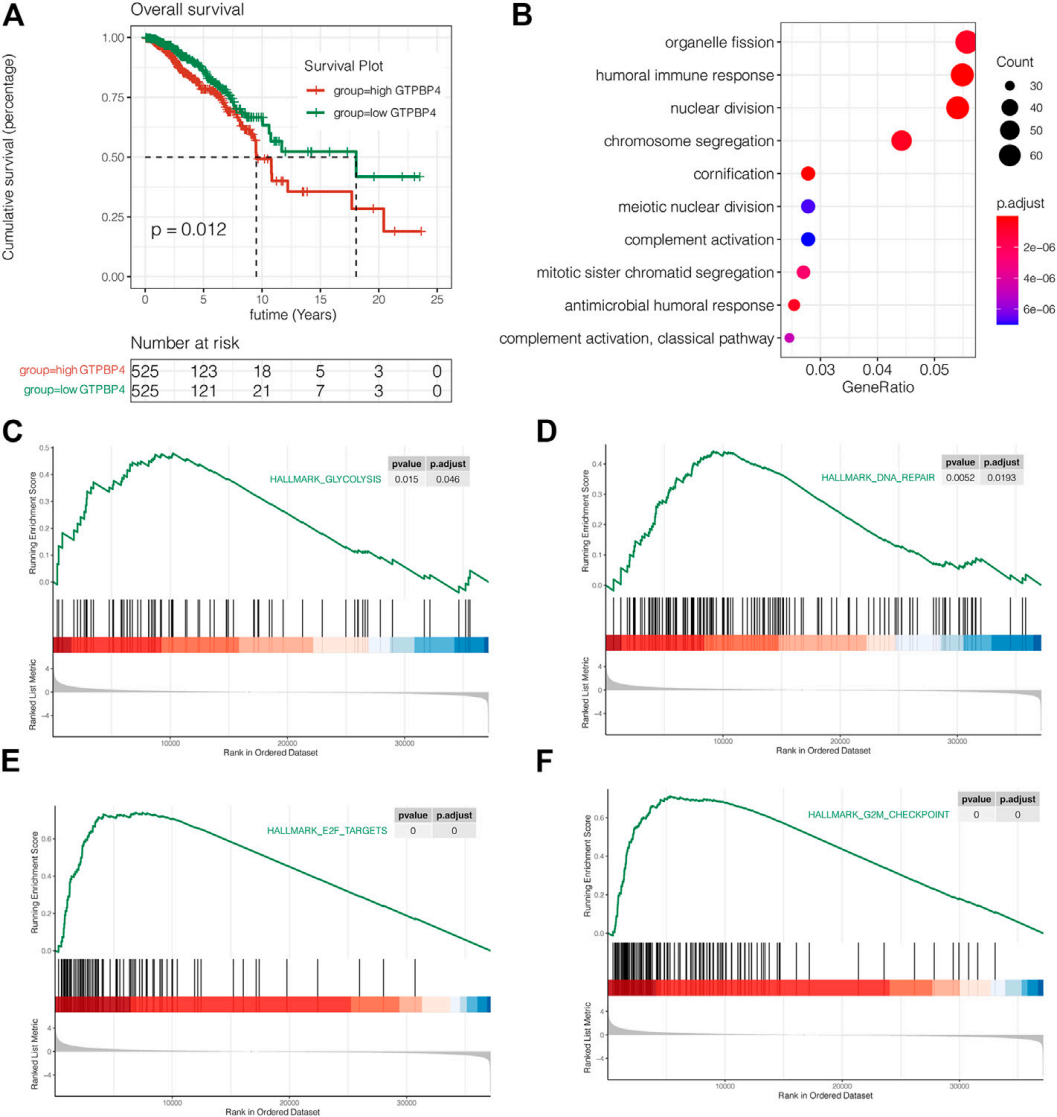
Survival analysis and enrichment analysis. **(A)** Survival analysis on TCGA cohort. The results showed that high expression of GTPBP4 significantly reduced the OS, overall survival of breast cancer patients (*p* = 0.012). **(B)** Gene ontology enrichment analysis showed that the differentially expressed genes between the high GTPBP4 group and low GTPBP4 were mainly enriched in organelle fission, humoral immune response and nuclear division **(C–F)** GSEA showed that glycolysis, DNA repair, E2F targets and G2M checkpoint pathways were significantly enriched in GTPBP4 high expression group.

Then there was a survival analysis. According to the median expression value of GTPBP4, TCGA breast cancer patients were separated into two groups: high expression and low expression. The difference in survival time between the two groups was then examined. The findings revealed that elevated GTPBP4 expression was linked to a poor breast cancer outcome (*p* = 0.012, Figure 6A). Following that, univariate and multivariate COX regressions were performed, with the results presented in Table 1 GTPBP4 was found to be an independent breast cancer predictive factor using COX regression. Table 2 shows the clinical information for these patients.

**TABLE 1:** Univariate and multivariate COX regressions.

Variable	GTPBP4low expression	GTPBP4over expression	X^2	*p*-value
Gender	436	435	4.528	0.033*
Female	434	426		
Male	2	9		
Age	436	435	5.029	0.025*
< = 65	299	328		
>65	137	107		
T	436	435	8.339	0.04*
T1	132	99		
T2	238	275		
T3	52	44		
T4	14	17		
N	436	435	3.647	0.302
N0	220	207		
N1	149	141		
N2	42	59		
N3	25	28		
M	436	435	2.956	0.086
M0	431	423		
M1	5	12		
Stage	436	435	5.381	0.146
Stage I	86	69		
Stage II	254	252		
Stage III	91	102		
Stage IV	5	12		

**TABLE 2:** Clinical information for these patients.

Characteristics	Total (N)	HR (95% CI) Univariate analysis	*P* value Univariate analysis	HR (95% CI) Multivariate analysis	*P* value Multivariate analysis
Gender	871		0.858		
Female	860	Reference			
Male	11	0.835 (0.1165.990)	0.858		
Age	871	1.035 (1.020–1.050)	<0.001	1.036 (1.021–1.052)	<0.001
T	871		<0.001		
T2	513	Reference			
T3	96	1.218 (0.737–2.010)	0.442	0.838 (0.452–1.553)	0.575
T1	231	0.692 (0.440–1.089)	0.111	1.004 (0.523–1.931)	0.989
T4	31	3.392 (1.887–6.098)	<0.001	1.362 (0.615–3.017)	0.446
N	871		<0.001		
N0	427	Reference			
N1	290	1.906 (1.270–2.861)	0.002	1.498 (0.891–2.517)	0.127
N2	101	2.470 (1.417–4.305)	0.001	1.579 (0.615–4.057)	0.342
N3	53	4.759 (2.581–8.777)	<0.001	1.956 (0.793–4.825)	0.145
M	871		<0.001		
M0	854	Reference			
M1	17	6.406 (3.597–11.406)	<0.001	4.087 (1.599–10.449)	0.003
Stage	871		<0.001		
Stage II	506	Reference			
Stage I	155	0.576 (0.322–1.031)	0.063	0.657 (0.267–1.619)	0.361
Stage III	193	1.853 (1.240–2.771)	0.003	1.561 (0.722–3.376)	0.257
Stage IV	17	6.884 (3.770–12.569)	<0.001		
GTPBP4	871	1.281 (1.006–1.631)	0.045	1.413 (1.100–1.815)	0.007

### Gene Ontology Enrichment Analysis and Gene Set Enrichment Analysis

Gene ontology enrichment analysis was used to find pathways that differed between the high and low GTPBP4 expression groups. The differently elevated genes in the two groups were mostly enriched in organelle fission, humoral immunological response, and nuclear division, according to the findings (Figure 6B). GSEA showed that glycolysis, DNA repair, E2F targets and G2M checkpoint pathways were significantly enriched in GTPBP4 high expression group (Figures 6C–F). This is significant for revealing the role of GTPBP4 in breast cancer.

### Analysis of Immune Microenvironment

**FIGURE 7:**
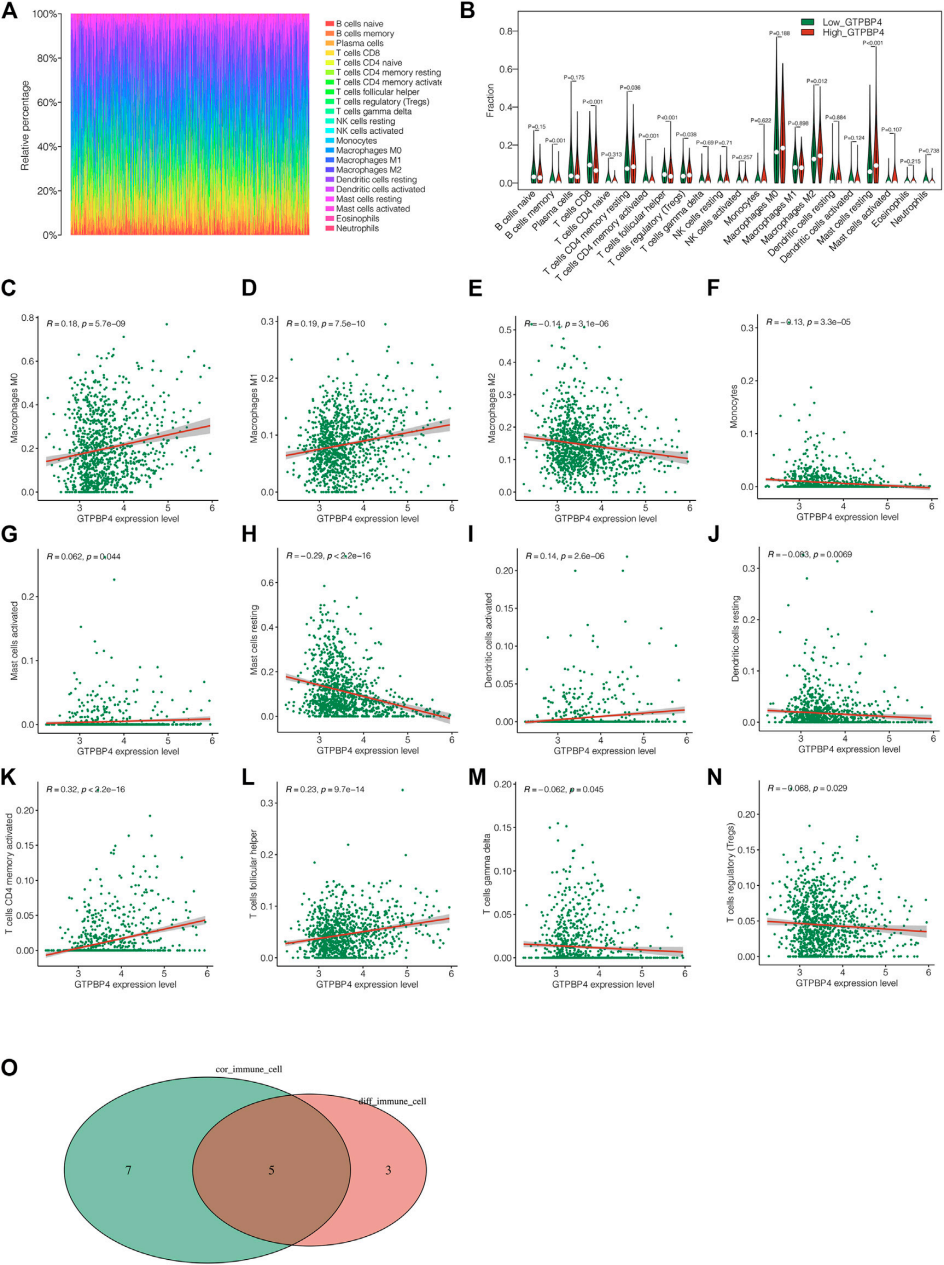
Analysis of immune microenvironment. **(A)** Immune landscape of breast cancer. We can see the proportion of different immune cell types. **(B)** The differences in immune cell infiltration levels between the high GTPBP4 expression group and the low GTPBP4 expression group, and 8 different immune cells were obtained **(C–N)** Correlation analysis showed that 12 kinds of immune cells were significantly correlated with GTPBP4 **(O)** Five most significant immune cell types were obtained by intersection of 8 different immune cell types and 12 related immune cell types.

The immunological landscape of breast cancer is depicted in Figure 7A. The presence of diverse immune cells is plainly obvious. Following that, we looked into the changes in immune cell infiltration levels between the high and low GTPBP4 expression groups, and discovered eight different immune cells (Figure 7B). Following that, correlation analysis revealed that GTPBP4 was significantly linked with 12 different types of immune cells (Figures 7C–N). Finally, the intersection of eight separate immune cell types and twelve related immune cell types yielded the five most important immune cell types (Figure 7O). T cells CD4 memory, T cells Follicular helper, T cells regulatory (Tregs), Macrophages M2, and Mast cells resting were identified. Supplementary Figure S2 showed the correlation analysis between GTPBP4 and tumor mutation load (TMB). It can be shown that the TMB level of the GTPBP4 high expression group was similarly high, and the TMB and GTPBP4 expression levels had a positive correlation. Supplementary Figure 3 depicted the relationship between GTPBP4 and the ESTIMATE score, Immune score, Stromal score, and tumor purity, highlighting GTPBP4’s function in the immune milieu.

### Construction of a Nomogram Based on GTPBinding Protein4 Expression

**FIGURE 8:**
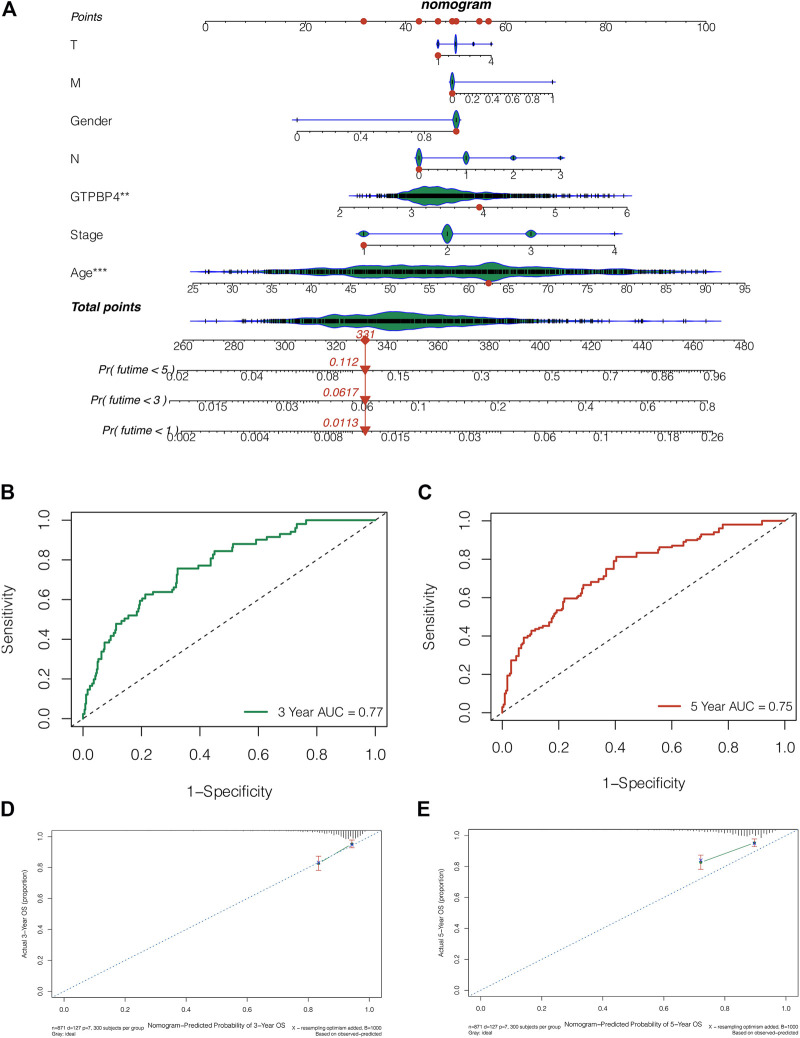
Nomogram was constructed to predict the prognosis of breast cancer patients. **(A)** The nomogram combining the expression of GTPBP4 and clinical characteristics. The 1-, 3-, and 5-years mortality rates of patient “TCGA-AR-A255” were 0.0113, 0.0617 and 0.112, respectively **(B,C)** The AUC values of this nomogram’s 3-years and 5-years ROC curves are 0.77 and 0.75, respectively **(D,E)** The 3-years and 5-years calibration curves showed that the nomogram can accurately predict the survival of breast cancer patients.

We created a nomogram integrating GTPBP4 expression and clinical features to further evaluate the survival of breast cancer patients. Patient “TCGA-AR-A255” had 1-, 3-, and 5-year death rates of 0.0113, 0.0617, and 0.112, respectively, as shown in Figure 8A. The AUC values of the 3-year and 5-year ROC curves for this nomogram were 0.77 and 0.75, respectively (Figure8B, 8c). The nomogram can accurately predict the survival of breast cancer patients, according to the 3-year and 5-year calibration curves (Figures 8D,E).

### GTPBinding Protein4 Is Overexpressed in Multiple Breast Cancer Cell Lines

**FIGURE 9:**
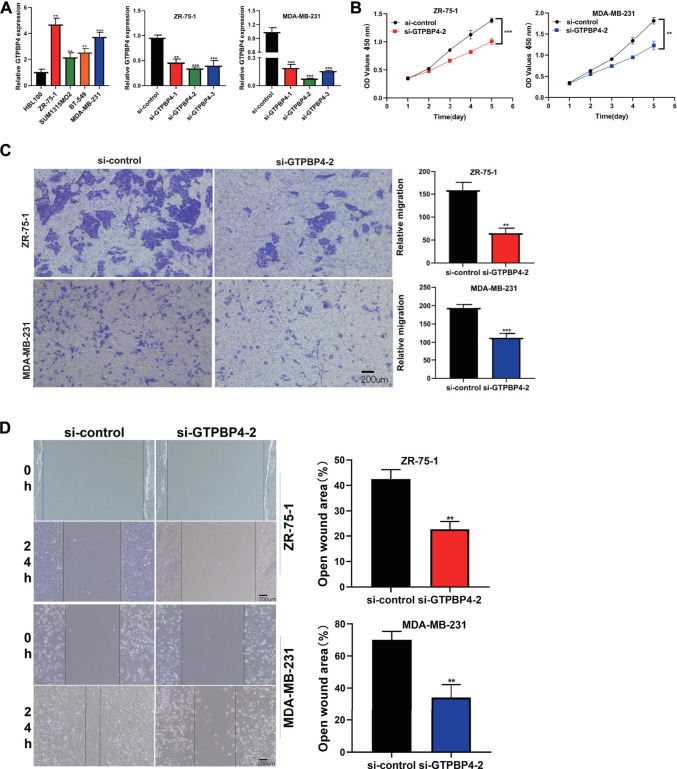
Cell assay was performed to verify the expression and function of GTPBP4 in breast cancer cell lines. **(A)** PCR assay showed that GTPBP4 expression was significantly up-regulated in four breast cancer cell lines ZR-75-1, SUM1315MO2, BT-549 and MDA-MB-231 compared with normal breast cell line HBL100. The expression of GTPBP4 was higher in ZR-75-1 and MDA-MB-231, so the gene knockdown experiment was carried out in ZR-75-1 and MDA-MB-231 cell lines. Three interfering RNAs were transfected, among which si-GTPBP4-2 had the highest transfection efficiency (*p < 0.05, **p < 0.01, ***p < 0.001). **(B)** CCK-8 assay showed that a decreased expression of GTBPB could inhibit the cell viability of ZR-75-1 and MDA-MB-231 breast cancer cell lines (**p < 0.01, ***p < 0.001). **(C)** Trasnwell assay showed that the migration capacity of ZR-75-1 and MDA-MB-231 breast cancer cell lines decreased significantly after GTBPB4 silencing (**p < 0.01, ***p < 0.001). **(D)**Scratch and Wound Healing assay showed a decreased speed of wound healing upon GTBPB4 gene knockdown (**p < 0.01).

The relative expression levels of GTPBP4 in Breast Cancer cell lines were quantified using qRT-PCR. In the cell lines ZR-75-1, SUM1315MO2, BT-549, and MDA-MB-231, we discovered that GTPB4 is overexpressed (Figure 9A). Because GTPBP4 expression was higher in the ZR-75-1 and MDA-MB-231 breast cancer cell lines, gene knockdown was performed in these two cell lines. With three different siRNA sequences, we inhibited the expression of GTPBP4 in ZR-75-1 and MDA-MB-231 breast cancer cell lines using RNAi technology. The knockdown potency of all three sequences was significant, although sequence 2’s knockdown effect was more desirable. As a result, it was employed in subsequent research.

#### Lowering the Expression Level of GTPBinding Protein4 Ameliorates Breast Cancer Cell Line Progression

We transfected GTPBP4 siRNA-2 into two different cell lines to explore the regulatory effect of GTBPB4 on BRCA *in vitro*. The CCK-8 experiment revealed that decreased GTBPB expression could limit the cell viability of the ZR-75-1 and MDA-MB-231 breast cancer cell lines (Figure 9B). The trasnwell assay consistently revealed that following GTBPB4 knockdown, the migratory capacity of two BRCA cell lines fell dramatically (Figure 9C). Furthermore, when the GTBPB4 gene was knocked out, the Scratch and Wound Healing assay revealed a slower rate of wound healing (Figure 9D). GTBPB4 seems to increase BRCA cell proliferation and migration, according to these data. All data were presented as the means ± SD of three independent experiments.

## Discussion

With the advent of the era of precision medicine, the treatment of breast cancer has also achieved rapid development (Xie et al., 2021c). At present, the treatment of breast cancer has formed a comprehensive treatment team composed of the radiology department, breast surgery department, plastic surgery department, oncology department, and rehabilitation department (Anastasiadi et al., 2017). The survival and well-being of breast cancer patients are increasing. However, unfortunately, breast cancer remains one of the most difficult diseases to treat in many countries, especially in less developed regions, due to the lack of education and medical care for patients (Woolston, 2015). Not only that, triple-negative breast cancer and advanced breast cancer also lack ideal treatment options (DeSantis et al., 2011). As the most common tumor in the world, it is time to explore its new biomarkers.

The importance of GTPBP4 in breast cancer was investigated in this study. To begin, we discovered that GTPBP4 expression was upregulated in breast cancer, which was confirmed by multiple cohorts. Second, we discovered that GTPBP4 may accurately diagnose breast cancer. GTPBP4 was found to be an independent breast cancer prognostic factor in subsequent survival analyses. The probable mechanism of GTPBP4 was discovered using GO enrichment analysis and GSEA enrichment analysis. The study of the immune microenvironment provides a foundation for understanding GTPBP4’s role in the tumor microenvironment.

Estrogen receptor (ER), progesterone receptor (PR), human epidermal growth factor receptor-2 (HER-2), and Ki-67 are the most well-known breast cancer indicators, and they are used to type breast cancer (Cedolini et al., 2014). Breast cancer can be classified into four types based on the above markers: luminal-A, luminal-B, HER2-overexpression, and triple-negative breast cancer (Roulot et al., 2016). This classification standard is a milestone in the history of breast cancer research. However, with the increase of patients and the progress of genomics, it has been found that this classification has some limitations in evaluating the prognosis of patients (Cedolini et al., 2014). In our study, GTPBP4, a novel biomarker, was discovered, which has significant significance for us to evaluate the prognosis of breast cancer patients and to understand the immune microenvironment. Immune reprogramming is one of the hallmarks of tumors (Xie et al., 2021d). Its specific mechanism, such as the discovery of immune checkpoints such as PD-1/PDL-1 and CTLA-4, has brought reference for the treatment of many solid tumors (Xie et al., 2021d). Immunotherapy has achieved promising outcomes in tumor types such as melanoma (Carreno et al., 2015; Cao et al., 2021; Xie et al., 2022). However, immunotherapy has progressed relatively slowly in breast cancer (Emens, 2018). Therefore, it is of great significance to explore the immune microenvironment of breast cancer. Our study provides an immune landscape for breast cancer, which can intuitively see the abundance of each immune cell. In addition, we also identified 5 immune cell types most closely related to GTPBP4, which is not only beneficial to our understanding of the function of GTPBP4 but also helpful to explore the immune microenvironment of breast cancer.

Overall, our findings can be used to aid in the detection and treatment of breast cancer. Our study, however, has several drawbacks. We don’t have enough relevant animal experiments to verify the results, but that will change in the future.

## Conclusion

Using a variety of approaches, we assessed the importance of GTPBP4 in breast cancer. The findings may be useful in the diagnosis and treatment of breast cancer.

## Data Availability

The original contributions presented in the study are included in the article/Supplementary Material, further inquiries can be directed to the corresponding author.

## Abbreviations

BRCA, breast cancer; GTPBP, GTP binding protein; HCC, hepatocellular carcinoma; TCGA, The Cancer Genome Atlas; GEO, Gene Expression Omnibus; GTEx, Genotypic Tissue Expression; FPKM, Fragments Per Kilobase Per Million; ROC, receiver operating characteristic; AUC, area under the curve; SMD, standardized mean difference; SROC, Summary Receiver Operator Characteristic; GO, Gene ontology; GSEA, gene set enrichment analysis; ER, estrogen receptor; PR, progesterone receptor; HER-2, human epidermal growth factor receptor-2.
