# Supplementary material for: Integrated Analysis of Genomic and Transcriptomic Profiles Identified the Role of GTP Binding Protein-4 (GTPBP4) in Breast Cancer
Source: Front Pharmacol. 2022 Jun 16;13:880445. doi: 10.3389/fphar.2022.880445 (PMC9243593; doi:10.3389/fphar.2022.880445)
Supplement: Supplementary file 2 [file Table1.docx]

| GTPBP4-1(Forward) | GGGAGTGCCAAGTACCCG |
| --- | --- |
| GTPBP4-1(Reverse) | GAGGTCTATGAAGTCCTTGGCG |
| GTPBP4-2(Forward) | CGGGAGTGCCAAGTACCCG |
| GTPBP4-2(Reverse) | AACGTGAGGTCTATGAAGTCCTTG |
| GAPDH(Forward) | GAACGGGAAGCTCACTGG |
| GAPDH(Reverse) | GCCTGCTTCACCACCTTCT |
