# Supplementary material for: Integrated Analysis of Genomic and Transcriptomic Profiles Identified the Role of GTP Binding Protein-4 (GTPBP4) in Breast Cancer
Source: Front Pharmacol. 2022 Jun 16;13:880445. doi: 10.3389/fphar.2022.880445 (PMC9243593; doi:10.3389/fphar.2022.880445)
Supplement: Supplementary file 5 [file Table2.docx]

| hGTPBP4 si-1 sense | GCGUAGUCUUGGUGUUGACAUTT |
| --- | --- |
| hGTPBP4 si-1 antisense | AUGUCAACACCAAGACUACGCTT |
| hGTPBP4 si-2 sense | AGACUCCAACCGUUAUUCAUATT |
| hGTPBP4 si-2 antisense | UAUGAAUAACGGUUGGAGUCUTT |
| hGTPBP4 si-3 sense | CUGACUGAGGAAGGUGUUAUUTT |
| hGTPBP4 si-3 antisense | AAUAACACCUUCCUCAGUCAGTT |
